# Efficacy and safety of enzyme replacement therapy with alglucosidase alfa for the treatment of patients with infantile-onset Pompe disease: a systematic review and metanalysis

**DOI:** 10.3389/fped.2024.1310317

**Published:** 2024-02-15

**Authors:** A. D. Dornelles, A. P. P. Junges, B. Krug, C. Gonçalves, H. A. de Oliveira Junior, I. V. D. Schwartz

**Affiliations:** ^1^ Faculty of Medicine, Universidade Federal do Rio Grande do Sul, Porto Alegre, Brazil; ^2^ Pediatric Service, Hospital de Clínicas de Porto Alegre, Porto Alegre, Brazil; ^3^ Faculty of Medicine, Universidade Federal do Rio Grande do Sul, Porto Alegre, Brazil; ^4^ Medical Genetics Service, Hospital de Clínicas de Porto Alegre, Porto Alegre, Brazil; ^5^ Nuclimed, Clinical Research Centre, Hospital de Clínicas de Porto Alegre, Porto Alegre, Brazil; ^6^ Health Technology Assessment Unit, Hospital Alemão Oswaldo Cruz, São Paulo, Brazil; ^7^ Department of Genetics, Universidade Federal do Rio Grande do Sul, Porto Alegre, Brazil

**Keywords:** enzyme replacement therapy, alglucosidase alfa, Pompe disease, glycogen storage disease type II, systematic review, meta-analysis

## Abstract

**Introduction:**

Pompe disease (PD) is a glycogen disorder caused by the deficient activity of acid alpha-glucosidase (GAA). We sought to review the latest available evidence on the safety and efficacy of recombinant human GAA enzyme replacement therapy (ERT) for infantile-onset PD (IOPD).

**Methods:**

We systematically searched the MEDLINE (via PubMed) and Embase databases for prospective clinical studies evaluating ERT for IOPD on pre-specified outcomes. Meta-analysis was also performed.

**Results:**

Of 1,722 articles identified, 16 were included, evaluating 316 patients. Studies were heterogeneous and with very low certainty of evidence for most outcomes. A moderate/high risk of bias was present for most included articles. The following outcomes showed improvements associated with alglucosidase alfa, over natural history of PD/placebo, for a mean follow-up of 48.3 months: left ventricular (LV) mass {mean change 131.3 g/m^2^ [95% confidence interval (CI) 81.02, 181.59]}, time to start ventilation (TSV) [HR 0.21 (95% CI: 0.12, 0.36)], and survival [HR 0.10 (95% CI: 0.05, 0.19)]. There were no differences between the pre- and post-ERT period for myocardial function and psychomotor development. Adverse events (AEs) after ERT were mild in most cases.

**Conclusion:**

Our data suggest that alglucosidase alfa potentially improves LV mass, TSV, and survival in IOPD patients, with no important safety issues.

**Systematic Review Registration:**

PROSPERO identifier (CRD42019123700).

## 1 Introduction

Pompe disease, also known as type II glycogenosis or acid maltase deficiency (OMIM 232300), is a rare lysosomal storage disorder that presents with a progressive neuromuscular involvement and is often fatal in the most severe forms. It is caused by the deficient activity of acid alpha-glucosidase (or acid maltase; EC 3.2.1.20), an enzyme responsible for glycogen degradation in the lysosomes (1). Deficient activity of this enzyme leads to an accumulation of glycogen within the lysosomes and cytoplasm of smooth, skeletal, and cardiac muscle. This accumulation ends up damaging cellular functioning and destroying cells, due to hypertrophy and rupture of lysosomes (2–7).

Infantile onset PD (IOPD), originally described by Pompe in 1932, involves patients with total acid alpha-glucosidase deficiency, with symptom onset at an average of between 1.6 and 2 months of life, which may even manifest in the uterus (8, 9). It is characterized by the presence of severe disease symptoms (3), such as generalized muscle weakness, cardiomegaly, and cardiac hypertrophy. The global incidence of PD is estimated at 1/40,000 new-borns (NBs), with 1/138,000 NBs for IOPD and 1/57,000 NBs for the late form. An ethnic influence is identified, as there is a high incidence of the disease in African American (1/12,000 NBs) and Chinese (1/40,000–1/50,000 NBs) patients (2, 4). Additionally, studies from newborn screening programs demonstrate higher incidence than reported previously: 1/25,200 NBs (IOPD and LOPD combined) in California, USA, as an example (10).

There is no curative treatment for PD. Currently, available treatment options are designed to address the mutant protein and consist of enzyme replacement therapy (ERT) with alglucosidase alfa (Myozyme™), a form of human acid alpha-glucosidase (GAA) produced by recombinant DNA technology in Chinese hamster ovary cells (3) or with avalglucosidase alfa-ngpt (Nexviazyme®). The recommended dosage regimen of alglucosidase alfa is 20 mg/kg body weight, administered every 2 weeks by intravenous (IV) infusion (11), although dosage can very accordingly to each patient. Furthermore, a new generation of rhGAA, cipaglucosidase alfa co-administered with an enzyme stabilizer, miglustat (12, 13), as well as gene therapy can be new alternatives for the treatment of these patients soon (14).

A previous systematic review (SR) on IOPD has already been published (15), without meta-analysis, and included only the study from Kishnani et al. (16). The authors concluded that this small trial provided no robust evidence about which dosing schedule of ERT is more effective and reinforces the need of a large RCT comparing different dosing schedules. They also affirm the need of standardizing the main clinical outcomes. Therefore, the impact of alglucosidase alfa treatment on key outcomes, such as cardiomyopathy and time to start ventilatory support (TSV), is still unclear. Within this context, the present SR with meta-analysis was designed to evaluate the effects of alglucosidase alfa ERT in IOPD.

## 2 Methodology

### 2.1 Information sources and search strategy

This study aimed to review the latest available evidence on the efficacy and safety of alglucosidase alfa ERT in IOPD. To guide the literature search, a structured “Patient, Intervention, Comparison and Outcome” (PICO) question was formulated as follows: “Is the use of alglucosidase alfa effective and safe as ERT in patients with IOPD?”. The MEDLINE (via PubMed) and Embase databases were searched for studies published before 25 April 2022, including terms for glycogen storage disease type II and alpha glucosidase (Supplementary Table S1), complemented by a manual search. The SR is reported as proposed by the PRISMA guidelines (17) and has been previously registered in the PROSPERO database (CRD42019123700).

### 2.2 Eligibility criteria and study selection

We planned to include only randomized clinical trials (RCT) and observational comparative studies in which ERT with alglucosidase alfa was used for the treatment of patients with IOPD. Other prospective study designs would be included (open-label and non-randomized trials, controlled or otherwise, including quasi-experimental designs) if the sample size was ≥5. *In vitro* studies or animal models, reviews, expert opinions, and retrospective studies were excluded. Unpublished work was covered by the identification of conference abstracts containing data deemed to be of interest. The final published articles were then included when available.

Studies that did not evaluate at least one of the eight outcomes of interest, defined *a priori* by a team of experts, were excluded. These outcomes were left ventricular (LV) mass, myocardial function [evaluated through LV ejection fraction (EF)], quality of life (QOL), survival, TSV in months, psychomotor development, decreased muscle tone, swallowing, and safety.

The selection stage was performed independently by two investigators (APPJ and CG), who assessed the abstracts retrieved during the search for eligibility. Decisions were compared, and articles deemed relevant were forwarded to two other investigators (ADD and BK) who independently extracted information on the characteristics of these studies (design, randomization methods, population of participants, interventions, and outcomes) using a standardized data collection form. The two investigators then took part in a consensus meeting. Any disagreement that remained was addressed by the intervention of a third investigator (HAOJ or IVDS). Finally, the references of the selected articles were hand-searched for potentially relevant studies not identified by the previous search strategies. When such information could not be retrieved, an email was sent to authors requesting non-reported data.

### 2.3 Data collection

Studies with data from the same population were excluded from meta-analyses. In these cases, the study with the largest sample (or, if both studies had the same sample size, that with the longest follow-up) was retained for analysis.

### 2.4 Statistical analysis

We summarized results using mean changes from baseline with 95% confidence intervals (CIs) for continuous outcomes. To incorporate follow-up time, we used incidence rates (IRs) with 95% CIs to summarize events. When not directly reported, the mean change from baseline and standard error estimates were approximated based on reported statistics (95% CI, *p*-values, median, and interquartile range). If needed, data conversion (e.g., confidence interval to standard deviation) would be performed (18).

Pairwise meta-analyses for dichotomous outcomes were analyzed using the Mantel-Haenszel method, with random model, and the DerSimonian-Laird estimator of tau^2^. Continuity correction of 0.5 in studies with zero cell frequencies were employed in all analyses. Quantitative assessment of time-dependent outcomes consisted of meta-analyses of studies reporting the hazard ratio (HR). Pooled analyses were performed on unadjusted HR with reported 95% confidence intervals (CIs) using an inverse of variance, random-effects model.

In addition to the qualitative analysis of methodological and clinical similarity of the studies, statistical analysis of heterogeneity was performed considering Higgins inconsistency analyses (*I*^2^) (19). Cochran's Q was considered statistically significant for heterogeneity if *P* < .10 (20). No threshold for statistical significance was used for the evaluation of clinical variables. Analyses were performed with R (version 3.2.3, R Core Team, The R Foundation, Vienna, Austria) and Review Manager (version 5.3.5 Copenhagen: The Nordic Cochrane Centre, The Cochrane Collaboration, 2014.).

### 2.5 Risk of bias of included studies

The risk of bias of included studies was evaluated with tools appropriate for each study designs: the Risk of Bias tool (RoB) 2.0 for RCTs and Risk of Bias in Non-randomized Studies of Interventions tool (ROBINS-I) for non-randomized studies of interventions (NRSI) (21, 22).

### 2.6 Certainty of evidence

Certainty of evidence of outcomes defined *a priori* was evaluated according to Gradings of Recommendation, Assessment, Development and Evaluation (GRADE) criteria (23–25). Assessment of certainty of the evidence for outcomes was performed independently by two investigators (ADD and HAOJ).

## 3 Results

**Figure 1:**
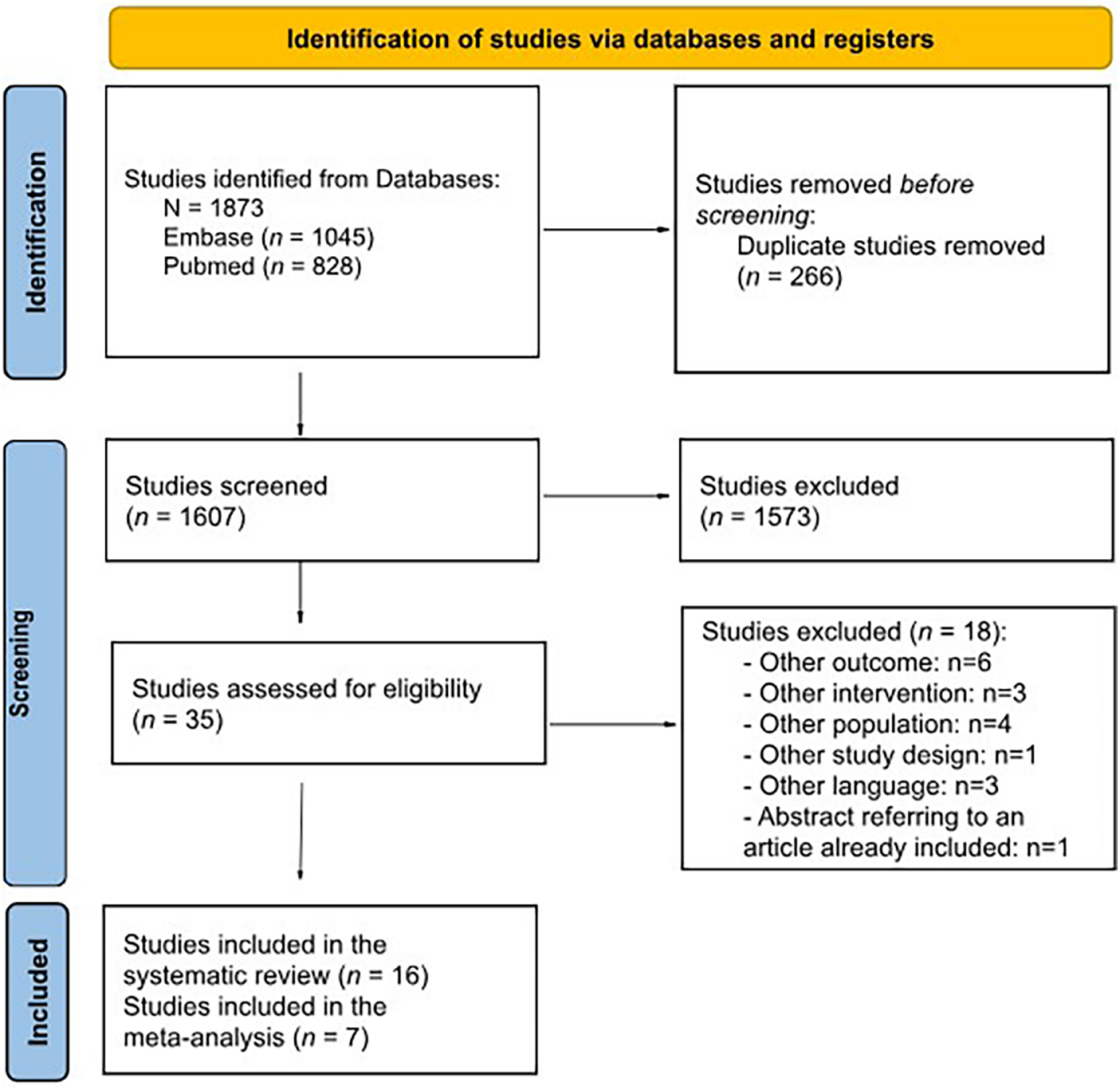
PRISMA flow diagram of search results.

The broad search strategy retrieved 1,722 references (797 from MEDLINE and 925 from Embase), of which 250 were duplicated. The titles and abstracts of 1,472 references were read, and 33 publications were selected for full-text evaluation. Of these, 16 were selected for eligibility, and 18 were excluded (the reason for deletion has been recorded and it is available in Supplementary Table S2). A PRISMA flow diagram of evidence selection is shown in Figure 1. Ultimately, 15 studies were identified for IOPD.

Among the included studies, there is one phase II open clinical trial (26), one open clinical trial (16) and its extension study (27), one case-control study, and all the others are NRSI. Studies that evaluated the outcomes defined *a priori* are described in Table 1. Our search did not retrieve any articles evaluating QOL, hypotonia, or swallowing disorder that matched the inclusion criteria; therefore, these outcomes could not be evaluated.

**Table 1:** Outcomes of interest defined *a priori* and studies that met the inclusion criteria.

Outcome	Number of articles	References
Left ventricular mass	11	(3, 16, 26–34)
Safety	9	(16, 26–30, 32, 35, 36)
Survival	9	(16, 26–29, 32, 35, 37, 38)
Time to start ventilatory support	8	(16, 26–29, 32, 34, 37)
Myocardial function	5	(16, 28, 30, 33, 37)
Psychomotor development	2	(16, 39)
Quality of life	0	
Hypotonia	0	
Swallowing disorder	0	

### 3.1 Characteristics of included studies

All included studies and their characteristics are described in Table 2. A total of 16 studies containing data from 316 patients were evaluated, for a mean follow-up time of 48.3 months. The mean age of starting ERT was 6.3 months, ranging from 0.1 to 43.1.

**Table 2:** Included studies and their characteristics.

Author	Patients (*n*; female)	Design	Age of onset of ERT -months—*μ* or median (range)	Intervention (alglucosidase alfa IV) and follow-up duration	Comparison	Follow-up (mean in months)	Patients on ventilation (*n*)	CRIM status (*n* of positive/total)
Barker et al. (30)	10; 2	NRSI	4 (1–10)	Unspecified dose for 36 months	–	36	–	8/10
Chen et al. (33)	9; 3	NRSI	1.8 (0.4–4.2)	20–40 mg/kg/2 weeks for 27.6 months (median)	–	27.6 (median)	–	N/A
Chien et al. (37)	6; 3	Case control	2.9 (0.4–14) (cases)	20 mg/kg/2 weeks for up to 40 months diagnosed with NBS	Alglucosidase alfa IV 20 mg/kg/2 weeks with clinical diagnosis (*n* = 10)	40	*I* = 0; *C* = 3 invasive	6/6
Chien et al. (32)	10; N/A	NRSI	0.5 (0.2–1.1)	20 mg/kg/2 weeks for up to 63 months (median)	Alglucosidase alfa IV 20 mg/kg/2 weeks with clinical diagnosis	63 (median)	Invasive = 5; non-invasive = 1	14/18
Ditters et al. (38)	116 cases, 8 controls; 58 cases, 2 controls	NRSI	3.3 (0.03–11.8)	20 mg/kg/2 weeks for up to 60.1 months	Alglucosidase alfa IV other doses	60.1	N/A	72/96 (controls N/A)
Kishnani et al. (26)	8; 4	Phase II open clinical trial and extension study	4.7 (2.7–14.6)	Initial phase 12.7 months: 10 mg/kg/wk.; Extension phase up to 38.2 months: 10–20 mg/kg/wk. or 20 mg/kg/2 weeks		38.2	Invasive = 1; deceased = 2	6/8
Kishnani et al. (16)	18; 7	Open ECR	5.3 (1.2–6.1)	20–40 mg/kg/2 weeks for 12.7 months	Untreated historical cohort	18	Invasive = 3; non-invasive = 3	15/18
Kishnani et al. (27)	18; 7	NRSI (extension study by Kishnani et al.; 2007)	5.3 (1.2–6.1)	20–40 mg/kg/2 weeks for 36 months	Untreated historical cohort	27.6 (median)	Invasive = 9	14/18
Levine et al. (3)	8; N/A	NRSI	4.7 (2.7–14.6)	10 mg/kg weekly for 12.7 months	–	13	N/A	N/A
Nagura et al. (35)	10; N/A	NRSI	13.9 (0.4–36.9)	20 mg/kg/2 weeks for 9 years	–	108	N/A	N/A
Nicolino et al. (28)	21; N/A	NRSI	13 (3.7–43.1)	20–40 mg/kg/2 weeks for 12.7 months	Untreated reference cohort	30 (median)	Invasive = 8; non-invasive = 0; deceased = 6	19/21
Spiridigliozzi et al. (39)	17; N/A	NRSI	5.2 (0.4–7.1)	20–40 mg/kg/2 weeks for 13 months	–	13	N/A	14/17
van Capelle et al. (34)	14; 7	NRSI	2.7 (0.1–8.3)	20 mg/kg/2 weeks or 40 mg/kg weekly for 57.6 months (median)	–	57.6 (median)	Invasive = 5	12/14
van Gelder et al. (31)	8; 4	NRSI	1.8 (0.1–4.6)	20 mg/kg/2 weeks for 63 months (median)	Alglucosidase alfa IV 40 mg/kg/week	43.6	*I *= 1/4; *C* = 0/4 (all invasive)	8/8
van Kooten et al. (36)	5; 3	NRSI	26.4 (13.2–40.8)	20–40 mg/kg/2 weeks for 12.4 years (median)	–	148.8 (median)	Invasive = 1; non-invasive = 3	N/A
Zhu et al. (29)	10; 6	NRSI	4.2 (1–6)	20 mg/kg every 2 weeks for up to 52 weeks		13		9/10
Total	316	–	6.3 (0.1–43.1)	–	–	48.3	–	197/244

ERT, enzyme replacement therapy; *μ*, mean; IV, intravenous; I, intervention; C, control; NBS, newborn screening; N/A, not available; NRSI, non-randomized study of intervention. Mean or median were used, as described in the included article.

### 3.2 Left ventricular mass

The LV mass was described in 11 studies, as shown in Table 1. The results of the included studies are detailed in Supplementary Table 2. There were three reports for the same study (16, 26, 27). Therefore, we only considered the first to be published as its data were completed (26). Kishnani et al. (16), Nicolino et al. (28) and Zhu et al. (29) showed the beneficial effect of ERT, with reductions in LV mass as follows: −106.6 g/m^2^ (16), −62.7% (28) and −227.60 ± 155.99 g/m^2^ (29), after ERT respectively.

**Figure 2:**
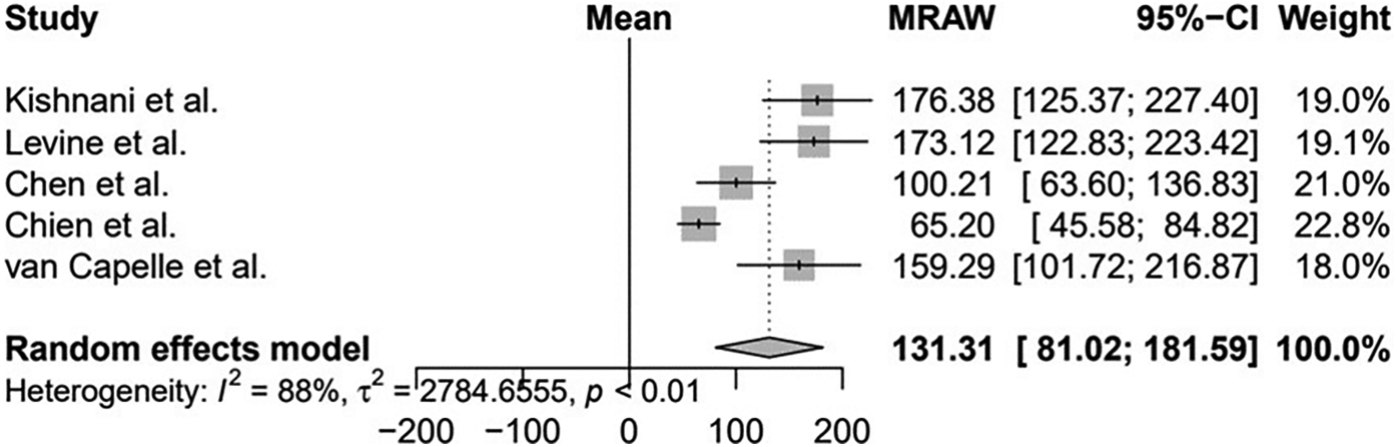
Evaluation of the left ventricular mass of patients with infantile-onset Pompe disease on enzyme replacement therapy with alglucosidase alfa. Weights are inverse-variance weights and are proportional to the contribution of each study to the summary estimate. *I*^2^ is the fraction of variance that is due to statistical heterogeneity and not chance. Tau^2^ denotes the between-study variance.

Furthermore, two studies with smaller samples showed stabilization of LV mass after treatment: Barker et al. (*n* = 5) (30) and van Gelder et al. (*n* = 8). Van Gelder et al. mentioned that one patient normalized LV mass after treatment, but the data were not detailed (31). For meta-analysis, we included 5/10 studies with comparable data and the results are shown in Figure 2, showing a significant reduction in LV mass after ERT, suggesting a benefit for LV mass, although with a high statistical heterogeneity (*I*^2 ^= 88%), mainly due to clinical heterogeneity of patients included.

### 3.3 Myocardial function

As an indicator of myocardial function, the ejection fraction (EF) was considered in four studies (Table 1). Nicolino et al. was the study with the largest sample to evaluate this outcome (*n* = 21) and showed improvement in myocardial function in 30.4% after ERT. However, comparison with other included studies should be cautious as authors used shortening fraction instead of EF (28). Data from other included studies showed stabilization of EF, with no significant differences before and after ERT (Supplementary Table S4).

### 3.4 Time to start ventilatory support

**Figure 3:**
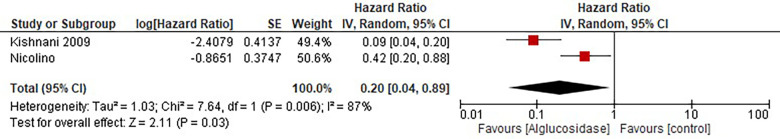
Evaluation of the time free of ventilation of patients with infantile-onset Pompe disease on enzyme replacement therapy with alglucosidase alfa. Weights are inverse-variance weights and are proportional to the contribution of each study to the summary estimate. *I*^2^ is the fraction of variance that is due to statistical heterogeneity and not chance. Tau^2^ denotes the between-study variance.

TSV was evaluated in eight studies (Table 1). Meta-analysis of 2/8 studies which described the time free of mechanical ventilation (MV) as hazard ratios was performed. Data are described in Figure 3. Complete data for TSV were described for 4/8 studies and suggest that ERT increases the time to start ventilation (Supplementary Table S5).

Chien et al. showed (37) and confirmed the finding with a follow-up study in 2015 (32) that time to start ventilation is prolonged after starting ERT in comparison to untreated cases (*P *< .001) (Supplementary Table S5). These studies also showed that the newborn screened population that started ERT earlier than the clinical cases population start ventilation even later (*P *< .001). Zhu et al. (29) showed that only 1/9 subjects used an invasive ventilator during the study. At the end of the study (13 months), all nine survivors were free from the use of any ventilators.

The study from Kishnani et al. (16) was not included in meta-analysis because its follow-up was included (27). The authors grouped the risk of death or invasive MV and this was reduced by 92% (HR 0.08, 95% CI: 0.03–0.21, *P *= .001) with ERT. Risk of death or any type of ventilation was reduced by 88% (HR 0.12, 95% CI: 0.05–0.29, *P *= .001). Three patients required MV before 18 months of age, with a survival rate without invasive MV of 88.9% (95% CI: 74.4%–100%). Non-invasive ventilation was required for another three patients, with a survival rate at 18 months without any type of ventilatory support of 66.7% (95% CI: 44.9%–88.4%). The follow-up study (27) corroborated its findings: 3/18 patients required MV at 18 months, 6/18 at 24 months, and 9/18 at 36 months, at the time of death or at the end of the study, whichever came first. The Kaplan–Meier ventilation-free survival rates at different ages were as follows: 66.7% (95% CI: 44.9%–88.4%) at 24 months and 49.4% (95% CI: 26%–72.8%) at 36 months. Thus, alglucosidase alfa reduced the risk of invasive ventilation or death by 91% (hazard ratio 0.09, 95% CI: 0.04–0.22), when compared to the historical control group (Figure 3).

### 3.5 Survival

**Figure 4:**
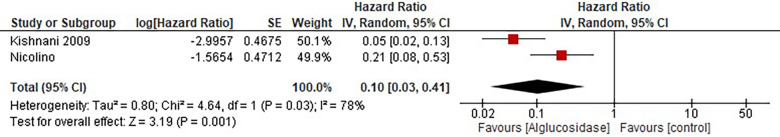
Evaluation of survival time of patients with infantile-onset Pompe disease on enzyme replacement therapy with alglucosidase alfa. Weights are inverse-variance weights and are proportional to the contribution of each study to the summary estimate. *I*^2^ is the fraction of variance that is due to statistical heterogeneity and not chance. Tau^2^ denotes the between-study variance.

Nine studies evaluated survival in IOPD and two were included in a meta-analysis (Figure 4). There was an increase in survival in all studies that evaluated this outcome. Meta-analysis suggests that the effect of ERT is potentially beneficial in patients' survival. However, there is uncertainty due to high statistical heterogeneity (*I*^2 ^= 78%).

Chien et al. (32) described a 100% survival rate (10/10) for patients treated for 63 months on average. This study was not included in the meta-analysis due to the incomplete reporting of data. Kaplan–Meier analysis indicated that the survival of patients diagnosed by newborn screening test was significantly better than untreated patients (*P *< .001) and late diagnosis patients (*P *= .03), data not shown. Kaplan–Meier analysis was also performed in the study by Chien et al. (37), with similar results: greater survival in patients from the early diagnosis (newborn screening) group (up to 40 months of follow-up, without deaths) compared to the untreated cohort (100% of deaths after 35 months of follow-up; *P *= .001) and without significant difference compared to patients treated with late diagnosis (65% mortality with a follow-up of 80 months; *P *= 0.48).

Nicolino et al. (28) compared the survival of patients starting treatment before and after 12 months of age with the survival of a historical control cohort without treatment, described in Kishnani et al. (26). Patients starting treatment before 12 months had a survival rate of 50% after 104 weeks of treatment (95% CI: 19%–81%; *n* = 10), against a survival rate of 9.2% (95% CI: 1.5 to 16.8%; *n* = 59) in the control cohort. Patients starting treatment after 12 months showed treatment survival of 91% (95% CI: 74%–100%; *n* = 11), against a survival rate of 45.5% (95% CI: 16%–75%; *n* = 11) after 104 weeks of follow-up. The reduction in the risk of death in treated patients compared to the historical cohort was 79% (*P *< .01).

In the study conducted by Kishnani et al. (26), two patients died, respectively, at 14.7 and 18.3 months, after 43 and 16 weeks of treatment, both due to respiratory failure. Kishnani et al. (16) evaluated 15 patients when they turned 18 months of age and compared them with a historical untreated cohort described in Kishnani et al. (40), in which only 1/61 patients reached this age. ERT reduced the risk of death by 99% (HR = 0.01; *P *= .001). Finally, Kishnani et al. (27), in 2009, presented data from 18 patients, of whom 17 reached 24 months of age, with a survival rate of 94.4% (95% CI: 83.9% to 100%) at this time and of 72% (95% CI: 47.9% to 96%) at 36 months. In Kishnani et al. (27), 7/18 patients reached 36 months of age. These data were also compared to the same historical controls, with survival at 24 and 36 months of 1.9% (95% CI: 0%–5.5%). Finally, the survival rate of Zhu et al. (29) was 100% (*n* = 9, 95% CI: 66.4%–100%).

### 3.6 Psychomotor development

As shown in Table 1, only two studies described this outcome. As an indicator of psychomotor development, the mental development index (MDI) was evaluated. MDI was considered for assessment because it was the sole measure which could be compared between studies and with data availability after the intervention (ERT). The results were evaluated in a very heterogeneous way and are described in Supplementary Table S6. However, like the study by Kishnani et al. (16), it did not present pre-treatment data. Therefore, it was not possible to assess its effect.

### 3.7 Safety

#### 3.7.1 Adverse events

The summary of safety results is described in Supplementary Table S7. Data for Nagura et al. (35) was not described as authors mixed IOPD and late-onset patients' data. Kishnani et al. (27) reported safety data for 18 patients undergoing treatment with ERT, of which 11 had 224 AEs, all mild or moderate in intensity, managed with reduced infusion speed or pause. The most common AEs were urticaria (*n* = 47 events), fever (*n* = 27), and desaturation (*n* = 24 events) and were most commonly reported in the 40 mg/kg dose group. Similar preliminary results had already been reported by Kishnani et al. in 2007 (16).

In Kishnani et al. (26), ERT was well tolerated, with all patients presenting at least one AE, mostly mild or moderate. The AEs described were as follows: skin rash, fever, changes in blood pressure or heart rate, and bronchospasm, all resolved with symptomatic treatment and by slowing or pausing the infusion. Nicolino et al. (28) described 42 AEs in 11 patients (52%), the most common being skin changes (13 events), vascular changes (10 events), and changes in vital signs (7 events), which were also resolved with symptomatic treatment and by slowing or pausing the infusion. The data, therefore, suggests that alglucosidase alfa is potentially safe as ERT in patients with IOPD, since most AEs are mild to moderate.

Zhu et al. (29) had at least one AE reported for each participant. All AEs identified in this study were treatment-emergent AE (TEAEs), and at least one serious TEAE (SAE) was reported for nine subjects (90%). The most frequently reported SAEs (≥20%) were pneumonitis (60%; *n* = 6), followed by pneumonia (30%; *n* = 3), respiratory tract infection (20%; *n* = 2), gastroenteritis rotavirus (20%; *n* = 2), and bronchitis (20%; *n* = 2).

#### 3.7.2 Anti-alglucosidase alfa antibodies

Nicolino et al. (28) reported that 19/20 patients (95%) developed anti-alglucosidase alfa antibodies (Ab), but none of these patients showed inhibitory activity during the entire follow-up. In the study by Kishnani et al. (27), 16/18 patients had high titers of Ab, but only 3/16 had levels above 20% of neutralizing Ab. Similar preliminary results had already been reported by Kishnani et al. in 2007 (16). Chien et al. (32) described the development of Ab in 9/10 patients, with no description of neutralizing Ab. In Kishnani et al. (26), all patients developed Ab during treatment, also without description of neutralizing Ab. Finally, in Barker et al. (30), there was also development of Ab in all patients, and one patient, who had high and sustained Ab titers, showed neutralizing Ab, with increased ventricular mass despite treatment. Cross reactive immunologic material (CRIM) status was available for 197/244 patients in included studies (Table 2). However, this status was very heterogeneously described for each outcome and, therefore, could not be assessed. Only Zhu et al. (29) described that an immune tolerance induction (ITI) regimen was used.

### 3.8 Risk of bias and quality of included studies

**Figure 5:**
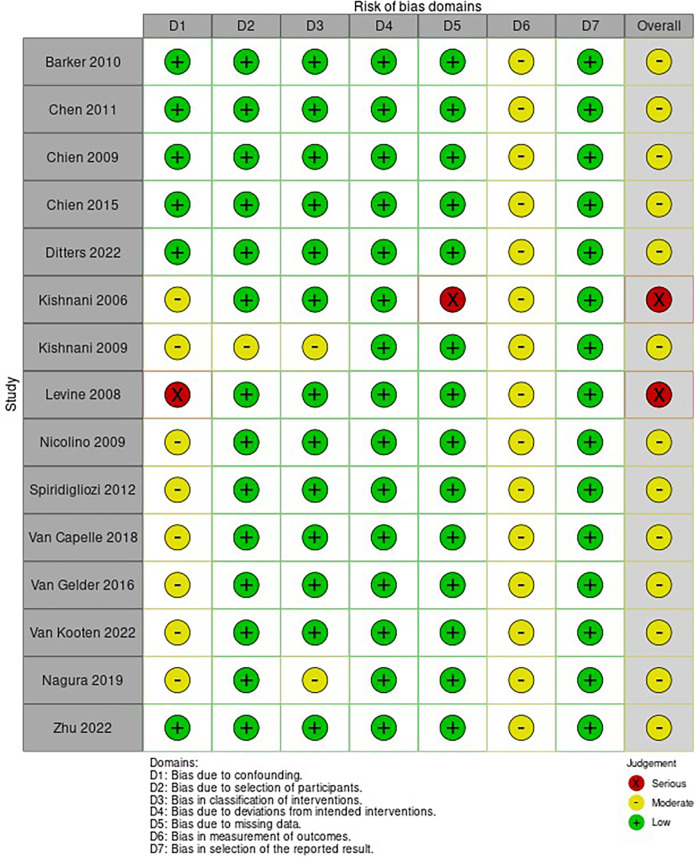
Risk of bias of included studies evaluated through the ROBINS-I tool. D6—The studies were not blinded. D1 Kishnani et al. (26)—Did not have an appropriate analysis method. D5 Kishnani et al. (6)—Outcome data were not available for all participants. D1 Kishnani et al. (27)—Patients with cardiac insufficiency were excluded. D1 Levine et al. (3)—Most patients had onset of symptoms after 1 year of age. D1 Nicolino—A patient was excluded due to discrepant cognitive function. D1 Spiridigliozzi et al. (39)—Patients with cardiac HF were excluded from the intervention group, but not from the control group. D1 Van Capelle—The duration of treatment was very discrepant between the groups. D1 Van Gelder—There was no statistical analysis and the two groups had different doses. D1 Van Kooten e Nagura—Did not differentiate between early and late forms in the analysis of outcomes (some patients started with symptoms after 1 year of age).

The risk of bias of the included NRSI is shown in Figure 5. Most included articles showed a moderate to serious risk of bias, independently of the study design, mainly due to confounding. Only one RCT was included (16) which was evaluated with serious risk of bias according to the R.o.B. 2.0, presenting some concerns in almost all domains but domain 3.

### 3.9 Certainty of evidence by outcomes

All outcomes were assessed for certainty of evidence, with low certainty for TSV and survival. All other outcomes had very low certainty of evidence, mainly due to the uncontrolled observational design of the included studies, with data from secondary outcomes. A full analysis is available in the Supplementary Table S8.

## 4 Discussion

IOPD is a rare, serious disease with no specific treatment available other than ERT. To the best of our knowledge, this is the first SR with meta-analysis to evaluate the effect of ERT on IOPD and included a total of 15 studies containing data from 316 patients. Patients were followed for a mean time of 48.3 months and the mean age of starting ERT was 6.3 months, ranging from 0.1 to 43.1. Among the outcomes evaluated for IOPD, a benefit for LV mass, TSV, and survival were seen in meta-analysis. Alglucosidase alfa appears to be safe in the population studied. Although the occurrence of AEs related to treatment or infusion are frequent, they are, in most cases, mild and easily treatable.

All studies had different ages, stages, durations of disease burden prior to start of ERT, and phenotypic manifestations. CRIM status was available in 12/16 included studies; however, it was very heterogeneously described for each outcome. However, CRIM status is known to have a big impact on outcomes and determines if an immune tolerance induction regimen is recommended concomitantly with ERT. Therefore, this needs to be taken into considerations while interpreting the results.

An SR on IOPD has already been published (15) and SR data for juvenile onset PD are available (41); none of them conducted a meta-analysis. The SR by Chen et al. (15) only included randomized trials and, therefore, included only Kishnani et al. (16), also considered in this SR. The authors concluded that this small trial provided no robust evidence for which dosing schedule of ERT is more effective and reinforced the need of a large RCT comparing different dosing schedules. They also affirmed the need of standardizing the main clinical outcomes, a limitation found in our SR as well (15).

Our results confirm a previously published study (42) and its follow-up (43) that evaluated cardiomyopathy, also indicating the beneficial effect of ERT with 4,000 L alglucosidase alfa for cardiac outcomes. Our study also indicates a beneficial effect of ERT on TSV, showing a reduction of 80% on the risk of being ventilated, with follow-up data up to 40 months, which can be considerable for this infantile form, in comparison with no treatment. A previous SR by our group for LOPD patients showed a beneficial effect of ERT on reducing time on ventilation (44).

Concerning survival, our study showed a beneficial effect of ERT for IOPD, indicating a reduction of 90% on the risk of death. A previous SR on juvenile patients (41) was not able to show any ERT effect for this population, as well as the previous SR by our group for LOPD patients (44), despite some effect on reducing mortality being reported by us. Recent data on IOPD included in this SR (38) showed that this improvement in survival can be even higher when patients are treated with high dosage ERT (40 mg/kg per week) when compared with patients treated with the standard dosage (20 mg/kg every other week); both studies included in meta-analysis (27, 28) did not separate data from different dosages, and therefore the effect described could be higher.

Regarding limitations, the included studies were highly heterogeneous, enrolling patients with different disease severities, and these are small, underpowered studies, with a high risk of bias. The certainty of the evidence was low, meaning the findings should be interpreted with caution, and it suggests that larger and more robust studies are encouraged. In addition, most included studies were non-randomized studies of interventions, with low methodological quality, no comparison group, or comparison with a historical cohort. Another challenge is incompletely reported data, as mentioned. Therefore, the findings of this review and meta-analysis should be interpreted with caution. Usually, data from secondary outcomes were poorly reported, as studies were not designed to measure them. In most cases, however, conclusions were made without showing full data and results. Proper reporting of data is essential.

Moreover, evaluating hypotonia, swallowing disorder, and pediatric quality of life, which are important outcomes for daily pediatric practice, must be addressed in future primary studies. Concerning psychomotor development, despite mentioned in few studies, the data suggest that the efficacy of ERT needs to be better evaluated for this outcome. One strength of the present study was that we included only prospective trials to avoid memory and selection bias. Prospective trials also have the advantage of collecting data according to the predefined outcomes of interest, which does not happen in retrospective trials. Also, of our best knowledge, this is the first SR with meta-analysis to evaluate the effect of ERT on IOPD and included a total of 15 studies containing data from 316 patients.

In conclusion, alglucosidase alfa potentially increases the survival rate, improves cardiac functioning, and may delay the start of ventilatory support in treated patients. The treatment is safe in the studied population, with generally mild adverse events. Further studies could evaluate the impact of the duration of follow-up, taking into consideration that the efficacy of ERT may present some secondary decline after 3 to 5 years of treatment (data for LOPD) (45).

## Data Availability

The original contributions presented in the study are included in the article/Supplementary Material, further inquiries can be directed to the corresponding author.
