# Supplementary material for: Efficacy and safety of enzyme replacement therapy with alglucosidase alfa for the treatment of patients with infantile-onset Pompe disease: a systematic review and metanalysis
Source: Front Pediatr. 2024 Feb 15;12:1310317. doi: 10.3389/fped.2024.1310317 (PMC10903525; doi:10.3389/fped.2024.1310317)
Supplement: Supplementary file 1 [file Table1.docx]

# Supplementary Tables

**Supplementary Table 1**. Search strategy for evidence in databases

| **Base** | **Search Strategy** |
| --- | --- |
| Medline  (via PubMed) | "Glycogen Storage Disease Type II"[Mesh] AND "alpha-Glucosidases"[Mesh] AND "humans"[MeSH] |
| Embase | 'glycogen storage disease type 2'/exp AND 'recombinant glucan 1, 4 alpha glucosidase'/exp OR 'recombinant glucan 1,4 alpha glucosidase' |

**Supplementary Table 2**. List of references excluded after reading the full text, according to the reason for exclusion.

| **Study design** |
| --- |
| Napolitano F, Bruno G, Terracciano C, Franzese G, Palomba NP, Scotto di Carlo F, Signoriello E, De Blasiis P, Navarro S, Gialluisi A, Melone MAB, Sampaolo S, Esposito T. Rare Variants in Autophagy and Non-Autophagy Genes in Late-Onset Pompe Disease: Suggestions of Their Disease-Modifying Role in Two Italian Families. Int J Mol Sci. 2021 Mar 31;22(7):3625. doi: 10.3390/ijms22073625. PMID: 33807278; PMCID: PMC8036926. |
| **Population** |
| Stockton DW, Kishnani P, van der Ploeg A, Llerena J Jr, Boentert M, Roberts M, Byrne BJ, Araujo R, Maruti SS, Thibault N, Verhulst K, Berger KI. Respiratory function during enzyme replacement therapy in late-onset Pompe disease: longitudinal course, prognostic factors, and the impact of time from diagnosis to treatment start. J Neurol. 2020 Oct;267(10):3038-3053. doi: 10.1007/s00415-020-09936-8. Epub 2020 Jun 10. PMID: 32524257; PMCID: PMC7501128. |
| Kuperus E, Kruijshaar ME, Wens SCA, de Vries JM, Favejee MM, van der Meijden JC, Rizopoulos D, Brusse E, van Doorn PA, van der Ploeg AT, van der Beek NAME. Long-term benefit of enzyme replacement therapy in Pompe disease: A 5-year prospective study. Neurology. 2017 Dec 5;89(23):2365-2373. doi: 10.1212/WNL.0000000000004711. Epub 2017 Nov 8. PMID: 29117951. |
| Nagura H, Hokugo J, Ueda K. Long-Term Observation of the Safety and Effectiveness of Enzyme Replacement Therapy in Japanese Patients with Pompe Disease: Results From the Post-marketing Surveillance. Neurol Ther. 2019 Dec;8(2):397-409. doi: 10.1007/s40120-019-00157-4. Epub 2019 Sep 26. PMID: 31559584; PMCID: PMC6858897. |
| Bar-Yoseph R, Mandel H, Mainzer G, Gur M, Tal G, Shalloufeh G, Bentur L. Cardiopulmonary exercise test to quantify enzyme replacement response in pediatric Pompe disease. Pediatr Pulmonol. 2018 Mar;53(3):366-373. doi: 10.1002/ppul.23830. Epub 2018 Jan 22. PMID: 29356433. |
| **Language** |
| Grosz Z, Várdi KV, Molnár JM. Tapasztalataink Pompe-betegségben terhesség alatt alkalmazott enzimpótló kezeléssel és az irodalom áttekintése [Pompe disease treated with enzyme replacement therapy in pregnancy]. Ideggyogy Sz. 2020 Sep 30;73(9-10):339-344. Hungarian. doi: 10.18071/isz.73.0339. PMID: 33035415. |
| Nicolino M. L'alglucosidase alpha, premier traitement pour la maladie de Pompe [Clinical development of acid alpha-glucosidase for the treatment of Pompe disease]. Presse Med. 2007 Mar;36 Spec No 1:1S100-7. French. PMID: 17546777. |
| Bereznai B, Trauninger A, György I, Szakszon K, Almássy Z, Pál E, Herczegfalvi A, Várdi Visy K, Illés Z, Molnár MJ. Pompe-kór fenotípusvariációi, kórlefolyása és az enzimpótló kezelés eredményei: hazai tapasztalatok [Clinical manifestations, course and outcome of enzyme replacement therapy in Hungarian patients with Pompe's disease]. Orv Hetil. 2011 Sep 25;152(39):1569-75. Hungarian. doi: 10.1556/OH.2011.29184. PMID: 21920843. |
| **Intervention** |
| Flotats-Bastardas M, Hahn A. New Therapeutics Options for Pediatric Neuromuscular Disorders. Front Pediatr. 2020 Nov 23;8:583877. doi: 10.3389/fped.2020.583877. PMID: 33330280; PMCID: PMC7719776. |
| Byrne, B., Colan, S., Kishnani, P., Foster, M., Sparks, S., Gibson, J., . . . Wang, R. (2022). Cardiac responses in paediatric Pompe disease in the ADVANCE patient cohort. Cardiology in the Young, 32(3), 364-373. doi:10.1017/S1047951121002079 |
| Klinge L, Straub V, Neudorf U, Schaper J, Bosbach T, Görlinger K, Wallot M, Richards S, Voit T. Safety and efficacy of recombinant acid alpha-glucosidase (rhGAA) in patients with classical infantile Pompe disease: results of a phase II clinical trial. Neuromuscul Disord. 2005 Jan;15(1):24-31. doi: 10.1016/j.nmd.2004.10.009. Epub 2004 Nov 26. PMID: 15639117. |
| **Outcome** |
| van Gelder CM, Hoogeveen-Westerveld M, Kroos MA, Plug I, van der Ploeg AT, Reuser AJ. Enzyme therapy and immune response in relation to CRIM status: the Dutch experience in classic infantile Pompe disease. J Inherit Metab Dis. 2015 Mar;38(2):305-14. doi: 10.1007/s10545-014-9707-6. Epub 2014 Apr 9. PMID: 24715333; PMCID: PMC4341007. |
| McDowell R, Li JS, Benjamin DK Jr, Morgan C, Becker A, Kishnani PS, Kanter RJ. Arrhythmias in patients receiving enzyme replacement therapy for infantile Pompe disease. Genet Med. 2008 Oct;10(10):758-62. doi: 10.1097/GIM.0b013e318183722f. PMID: 18813140; PMCID: PMC3612889. |
| Chien YH, Lee NC, Peng SF, Hwu WL. Brain development in infantile-onset Pompe disease treated by enzyme replacement therapy. Pediatr Res. 2006 Sep;60(3):349-52. doi: 10.1203/01.pdr.0000233014.84318.4e. Epub 2006 Jul 20. PMID: 16857770. |
| Yang CC, Chien YH, Lee NC, Chiang SC, Lin SP, Kuo YT, Chen SS, Jong YJ, Hwu WL. Rapid progressive course of later-onset Pompe disease in Chinese patients. Mol Genet Metab. 2011 Nov;104(3):284-8. doi: 10.1016/j.ymgme.2011.06.010. Epub 2011 Jun 22. PMID: 21757382. |
| Schneider I, Hanisch F, Müller T, Schmidt B, Zierz S. Respiratory function in late-onset Pompe disease patients receiving long-term enzyme replacement therapy for more than 48 months. Wien Med Wochenschr. 2013 Jan;163(1-2):40-4. doi: 10.1007/s10354-012-0153-5. Epub 2012 Nov 19. PMID: 23160972. |
| Schneider I, Hanisch F, Müller T, Schmidt B, Zierz S. Respiratory function in late-onset Pompe disease patients receiving long-term enzyme replacement therapy for more than 48 months. Wien Med Wochenschr. 2013 Jan;163(1-2):40-4. doi: 10.1007/s10354-012-0153-5. Epub 2012 Nov 19. PMID: 23160972. |
| **Abstract referring to an article already included** |
| Thurberg BL, Carlier P, Kissel JT, Schoser B, Pestronk A, Barohn RJ, Goker-Alpan O, Mozaffar T, Pena LD, Simmons Z, Straub V, Young P, Shafi R, Bjartmar C, van der Ploeg A. A Phase 4 Prospective Study in Patients with Adult Pompe Disease Treated with Alglucosidase Alfa. J Neuromuscul Dis. 2015;2(s1):S72-S73. PMID: 27858658. |

**Supplementary Table 3**. Evaluation of the effect of Enzyme Replacement Therapy in left ventricular mass associated with Early Onset Pompe Disease

| **Study** | **Patients (n); >12m** | **Outcome** | **Basal period**  **mean (SD)** | **Post ERT**  **mean (SD)** | **ERT Effect** | **Methodological limitations** |
| --- | --- | --- | --- | --- | --- | --- |
| **Kishnani *et al.* (2006)** | 8 (4 fem); 1 | LV mass (g/m²) | 266.9 ± 64.4 | 83.7 ± 25.3 | Average improvement rate of 68.7% | Absence of a control group, open study, no evaluation before and after ERT. |
| **Kishnani *et al.* (2007)** | 18 (7 fem); 0 | LV mass (g/m²) | 193.4 | 86.8 | - | Absence of a control group, open study, no evaluation before and after ERT. |
|  |  | LV mass (Z score) | 7.1 | 3.3 | - |  |
| **Levine *et al.* (2008)** | 8 (4 fem); 1 | LV mass (g/m²) | 191 (range 157 – 565) | 87 (range 54 – 124) | p < 0.001 (mean change) | Absence of a control group. |
| **Kishnani *et al.* (2009)** | 18 (7 fem); 0 | LV mass (Z score) | 7.1 | 2.0 | - | Absence of a control group, no evaluation before and after ERT. |
| **Nicolino *et al.* (2009)** | 21 (11 fem); NA | LV mass (Z score) | 6.5±2.6 | 0.9±0.8 | % variation after TRE =5.3% | Absence of a control group. |
|  |  | LV mass (g/m²) | 193.8±98.5 | 53.1±13.9 | % variation after TRE = -62.7% |  |
| **Barker *et al.* (2010)** | 5 (NA); 0 | LV mass (g/m²) | 69.9 | 71 | p= N.S.  (mean change) | Absence of a control group, small sample size. |
| **Chen *et al.* (2011)** | 9 (3 fem); 0 | LV mass (g/m²) | 156.5 | 72.5 | p<0.01  (mean change) | Absence of a control group. |
| **Chien *et al.* (2015)** | 10 (NA); 0 | LV mass (g/m²) | 132.4 | 67.2 | - | Absence of a control group, no evaluation before and after ERT. |
| **van Gelder *et al.* (2016)** | 8 (4 fem); 0 | LV mass (g/m²) | normal in 3/4 patients | normal in 4/4 patients | - | No pre and post ERT evaluation, small sample size. |
| **van Capelle *et al.* (2018)** | 14 (7 fem); 0 | LV mass (g/m²) | 226 | 70.8 | - | Absence of a control group, no evaluation before and after ERT. |
|  |  | LV mass (Z score) | 7 | 0.3 | - |  |
| **Zhu *et al.* (2022)** | 10 (6 fem); 0 | LV mass (g/m²) | 298.02 ± 178.43 | 70.59 ± 39.93 | - | Absence of a control group. |
|  |  | LV mass (Z score) | 16.63 ± 5.17 | 4.30 ± 2.78 | - |  |

*>12m=number of patients older than 12 months. Fem=female. SD=standard deviation. ERT=Enzyme Replacement Therapy. C=control. CI=confidence interval. LV=left ventricular. NA=not available.

**Supplementary Table 4**. Evaluation of the effect of Enzyme Replacement Therapy on myocardial function assessed with LV ejection fraction in patients with early Pompe Disease.

| **Study** | **Patients (n); >12m*** | **Outcome** | **Basal period**  **µ±sd (%)** | **After ERT**  **µ±sd (%)** | **ERT Effect** | **Methodological limitations** |
| --- | --- | --- | --- | --- | --- | --- |
| **Kishnani *et al.* (2007)** | 18 (7 fem); 0 | LV ejection fraction | 51.2 | 54.9 | - | Absence of a control group, open study, no pre- and post-ERT evaluation. |
| **Nicolino *et al.* (2009)** | 21 (11 fem); NA | LV shortening fraction | 35.6±12.1 | 43.8  ±6.5 | % variation after TRE = 30.4% | Absence of a control group. |
| **Barker *et al.* (2010)** | 5 (NA); 0 | LV ejection fraction | 51 | 55 | p=0.69 (mean change) | Absence of a control group, small sample size. |
| **Chen *et al.* (2011)** | 9 (3 fem); 0 | LV ejection fraction | 73.1 | 72.4 | not significant p | Absence of a control group |
| **Chien *et al*. (2009)** | 6 (3 fem); 0 | LV ejection fraction | NA | NA | No change from baseline | Open study, no pre- and post-ERT evaluation |
| **Zhu *et al.* (2022)** | 10 (6 fem); 0 | LV ejection fraction | 57.9±13.3 | NA | NA | Absence of a control group, open study, no pre- and post-ERT evaluation. |

*>12m= number of patients older than 12 months. Fem=female. SD=standard deviation. ERT=Enzyme Replacement Therapy. CI=confidence interval. LV=left ventricular. NA=not available.

**Supplementary Table 5**. Evaluation of Time to Start Ventilatory Support in patients with early Pompe Disease.

| **Study** | **Patients (n); >12m*** | **I or pre ERT**  **(mean)** | **C or after ERT (mean)** | **ERT Effect** | **Methodological limitations** |
| --- | --- | --- | --- | --- | --- |
| **Kishnani *et al.* (2006)** | 8 (4 female); 1 | SV= 8 patients | 11.3 months for IV (n=1); NIV or SV: n=5; 2 deaths | - | Absence of a control group, open study. |
| **Kishnani *et al.* (2007)** | 18 (7 female); 0 | I: Between 9.1 and 15 months for IV (n=3); between 7.8 and 14.8 for NIV (n=3); SV: n=12 | C: Between 4 and 16 months for IV or NIV. | Survival without IV or NIV (Kaplan-Meier): 66.7% (44.9% to 88.4%). | Open study. |
| **Kishnani *et al.* (2009)** | 18 (7 female); 0 | I: Between 9.1 and 29.6 months for IV (n=9); SV: n=9. | C: Between 4 and 16 months for IV or NIV. | Survival without IV (Kaplan-Meier): 49.4% (26% to 72.8%). | - |
| **Nicolino *et al.* (2009)** | 21 (11 female); NA |  |  | HR: 0.421 (0.202-0.876); p=0.0207 | Absence of a control group. |
| **Chien *et al*. (2009)** | 6 cases (3 female); 1 | I: 40 months (full follow-up) | C: 8 to 18 months | p=0.008 (mean difference) | Small sample size. |
| **Chien *et al.* (2015)** | 10 (NA); 0 | I: 63 months (full follow-up) | C: 8 to 42 months | p<0.001 (mean difference) | - |
| **van Capelle *et al.* (2018)** | 14 (7 female); 0 |  | 21.6 months for VI; NIV or SV=9. | - | Absence of a control group. |

*>12m= number of patients older than 12 months. I=intervention. SD=standard deviation. ERT=Enzyme Replacement Therapy. C=control. CI=confidence interval. SV=survival. IV=invasive ventilation. NIV=non-invasive ventilation. HR=hazard ratio. NA=not available.

**Supplementary Table 6**. Evaluation of the effect of Enzyme Replacement Therapy for early Pompe disease on psychomotor development using the MDI scale in early PD.

| **Study** | **Patients (n)** | **Pre ERT**  **n or µ** | **After ERT**  **n or µ** | **Methodological limitations** |
| --- | --- | --- | --- | --- |
| **Kishnani *et al.* (2007)** | 17 (NA); 0 | Data not available | 9/17 (53%) normal MDI (85 to 115)  4/17 (23%) slightly late (70-84)  4/17 (23%) significantly late (<69) | Absence of a control group; open study; without adequate pre and post ERT evaluation. |
| **Spiridigliozi *et al.* (2012)** | 16 (NA); 0 | MDI score adjusted/not adjusted:  - Good answer (n=12): 86.3/80.1  - Partial answer (n=4): 60.8/60.8  - Total (n=16): 79.9/75.3 | MDI score adjusted/not adjusted:  - Good answer (n=13): 89.8/84.7  - Partial answer (n=3): 52.3/51.3  - Total (n=16): 82.8/78.4 | Absence of a control group; without adequate pre and post ERT evaluation. |

ERT=Enzyme Replacement Therapy. MDI=mental development index. NA=not available.

**Supplementary Table 7**. Safety assessment of Enzyme Replacement Therapy for patients with Early Onset Pompe Disease

| **Study** | **Intervention (IV alglucosidase alfa)** | **Outcome** | **Total patients (n)** | **Number of Events** |
| --- | --- | --- | --- | --- |
| **Kishnani *et al.* (2006)** | Initial phase: 10 mg/kg/wk  Extension phase: 10-20 mg/kg/wk or 20 mg/kg/2 wk | SAE | 8 | 0 |
|  |  | Patients with IAR | 8 | 7 |
|  |  | Ab+ | 8 | 8 |
| **Kishnani *et al.* (2007)** | 20-40 mg/kg/2 week | Death | 18 | 1 |
|  |  | SAE | 18 | 0 |
|  |  | IAR | 11 | 164 |
|  |  | patients with IAR | 18 | 11 |
|  |  | Ab + | 18 | 16 |
|  | 20 mg/kg/2 week | IAR | 5 | 41 |
|  | 40 mg/kg/2 week | IAR | 6 | 123 |
| **Kishnani *et al.* (2009)** | 20-40 mg/kg/2 week | SAE | 18 | 0 |
|  |  | IAR | 11 | 224 |
|  |  | patients with IAR | 18 | 11 |
|  |  | Ab + | 18 | 16 |
|  | 20 mg/kg/2 week | IAR | 5 | 47 |
|  | 40 mg/kg/2 week | IAR | 6 | 177 |
| **Chien *et al.* (2015)** | 20 mg/kg/2 week | Ab + | 10 | 9 |
| **Nicolino *et al.* (2009)** | 20-40 mg/kg/2 week | IAR | 11 | 42 |
|  |  | Patients with IAR | 21 | 11 |
|  |  | Death | 21 | 6 |
|  |  | Ab + | 20 | 19 |
| **Barker *et al.* (2010)** | Unspecified dose | Ab + | 10 | 10 |
| **Van Kooten *et al*., (2022)** | 20 mg/kg/2 week | Patients with IAR | 22 | 4 |
|  |  | IAR | 4 | 59 |
| **Zhu *et al*. (2022)** | 20 mg/kg/2 week | Patients with IAR | 10 |  |

IV=intravenous. AE=adverse event. SAE=serious adverse event. IAR=infusion-associated reaction. Ab+=presence of anti-alglucosidase alfa antibodies.

**Supplementary Table 8**. GRADEpro Certainty assessment

| 1. **Alglucosidase alfa compared to placebo or natural history for Early Onset Pompe Disease** | | | | | | | | | | | |
| --- | --- | --- | --- | --- | --- | --- | --- | --- | --- | --- | --- |
| **Certainty assessment** | | | | | | | **Summary of findings** | | | | |
| **Participants (studies) Follow-up** | **Risk of bias** | **Inconsistency** | **Indirectness** | **Imprecision** | **Publication bias** | **Overall certainty of evidence** | **Study event rates (%)** | | **Relative effect (95% CI)** | **Anticipated absolute effects** | |
|  |  |  |  |  |  |  | **With placebo or natural history** | **With alglucosidase alfa** |  | **Risk with placebo or natural history** | **Risk difference with alglucosidase alfa** |
| **General Survival** | | | | | | | | | | | |
| 207 (2 observational studies) | serious^a^ | very serious^b^ | not serious | not serious | publication bias strongly suspected very strong association^g^ | ⨁⨁◯◯ Low | 141/168 (83.9%) | 11/39 (28.2%) | **HR 0.10** (0.03 to 0.41) | 839 per 1.000 | **672 fewer per 1.000** (from 786 fewer to 312 fewer) |
| **Ventilation-Free Survival** | | | | | | | | | | | |
| 207 (2 observational studies) | serious^a^ | very serious^b^ | not serious | not serious | publication bias strongly suspected very strong association^g^ | ⨁⨁◯◯ Low | 161/168 (95.8%) | 23/39 (59.0%) | **HR 0.20** (0.04 to 0.89) | 958 per 1.000 | **488 fewer per 1.000** (from 839 fewer to 17 fewer) |

| 1. **Alglucosidase alfa compared to placebo or natural history for Early Onset Pompe Disease** | | | | | | | |
| --- | --- | --- | --- | --- | --- | --- | --- |
| **Certainty assessment** | | | | | | |  |
| **Participants (studies) Follow-up** | **Risk of bias** | **Inconsistency** | **Indirectness** | **Imprecision** | **Publication bias** | **Overall certainty of evidence** |  |
|  |  |  |  |  |  |  |  |
| **Cardiomyopathy (follow-up: median 36 months; assessed with: LV mass (Z score and g/m²))** | | | | | | | |
| 121 (11 observational studies) | serious^a^ | very serious^b^ | serious^c^ | serious^d^ | publication bias strongly suspected strong association^e^ | ⨁◯◯◯ Very low |  |
| **Myocardial Function (follow-up: median 30.3 months; assessed with: LV Ejection fraction)** | | | | | | | |
| 59 (5 observational studies) | serious^a^ | serious^b^ | serious^c^ | serious^d^ | publication bias strongly suspected^e^ | ⨁◯◯◯ Very low |  |
| **Neuropsychomotor development (follow-up: median 15.5 months; assessed with: Mental development index)** | | | | | | | |
| 33 (2 observational studies) | very serious^a^ | very serious^f^ | serious^c^ | serious^d^ | publication bias strongly suspected^e^ | ⨁◯◯◯ Very low |  |
| **Safety (follow-up: median 36 months; assessed with: Adverse events)** | | | | | | | |
| 128 (9 observational studies) | very serious^a^ | serious^b^ | serious^c^ | serious^d^ | publication bias strongly suspected^e^; dose response gradient | ⨁◯◯◯ Very low |  |

S6.a) Studies evaluated by hazard ratio. b) Studies evaluated in a narrative way. CI: confidence interval; HR: hazard Ratio. Explanations: a. Open studies without control group; b. High heterogeneity between studies, without controlling for confounding; c. Data from secondary outcomes; d. No measure of effect assessed; e. Data obtained with manufacturer support; f. No controlling for confounding; g. not paired controls, data obtained with manufacturer support.
